# Transferrin-Pep63-liposomes accelerate the clearance of Aβ and rescue impaired synaptic plasticity in early Alzheimer’s disease models

**DOI:** 10.1038/s41420-021-00639-1

**Published:** 2021-09-21

**Authors:** Xiu Yang, Xu Li, Le Liu, Yuan-Hao Chen, Yue You, Yin Gao, Yue-Ying Liu, Li Yang, Kun Tong, Di-Shi Chen, Jing-Ru Hao, Nan Sun, Zi-Ming Zhao, Can Gao

**Affiliations:** 1 NMPA Key Laboratory for Research and Evaluation of Narcotic and Psychotropic Drugs, Jiangsu Province Key Laboratory of Anesthesiology, Jiangsu Province Key Laboratory of Anesthesia and Analgesia Application, Xuzhou Medical University, 221004 Xuzhou, Jiangsu China; 2 Jiangsu Province Key Laboratory of New Drug Research and Clinical Pharmacy, Xuzhou Medical University, 221004 Xuzhou, Jiangsu China

**Keywords:** Cognitive ageing, Drug delivery

## Abstract

Alzheimer’s disease (AD) is characterized by aberrant accumulation of extracellular β-amyloid (Aβ) peptides in the brain. Soluble Aβ oligomers are thought to be the most neurotoxic species and are correlated with cognitive dysfunction in early AD. However, there is still no effective treatment so far. We determined that Pep63, a small peptide, had a neuroprotective effect on synaptic plasticity and memory in our previous study. Here, we developed novel and multifunctional liposomes targeting both Aβ oligomers and fibrils based on a liposome delivery system. Transferrin-Pep63-liposomes (Tf-Pep63-Lip), possessing the ability for blood-brain barrier targeting, were also incorporated with phosphatidic acid (PA) and loaded with neuroprotective Pep63. We discovered that administration of Tf-Pep63-Lip could significantly reduce the Aβ burden in the hippocampus, and improve cognitive deficits in 6-month-old APP/PS1 mice in the Morris-Water maze task and fear-conditioning test with the combined effects of PA and Pep63. Tf-Pep63-Lip could capture Aβ oligomers or fibrils and then facilitated microglial chemotaxis nearby for clearance. Simultaneously, Tf-Pep63-Lip hindered Aβ1-42 aggregation and disaggregated Aβ1-42 assembly due to multivalent PA-Aβ. Pep63 effectively inhibited the binding between EphB2 and Aβ oligomers after release from liposomes and rescued NMDA receptors trafficking, the basis of synaptic plasticity. No side effects were observed in either APP/PS1 or wild-type mice, indicating that Tf-Pep63-Lip might be safe under the dosing regimen used in our experiment. Taken together, our results suggested that Tf-Pep63-Lip may serve as a safe and efficient agent for AD combination therapy.

## Introduction

Alzheimer’s disease (AD) is a common neurodegenerative disease characterized by progressive memory loss, cognitive decline, executive dysfunction, and so on [1, 2]. Although scientists have studied AD since 1906 [3], no medicine has been proven effective in reversing the disorder in the clinic so far [4–8]. The factors that contribute to relatively limited advancements in AD therapy are the complex pathogenesis with aging [9–13] and the tight blood-brain barrier (BBB). The initial factor that triggers the pathological cascade of AD is unknown, while the progressive and complex interactions of age-related risk factors further make interventions for AD a daunting task. Therefore, early preventive interventions and a combination of different anti-dementia drugs are believed to be promising efforts. Notably, the safety of AD drugs should be taken into consideration first due to the aging and poorly tolerant patients, who are more likely to suffer from complications [14].

The main obstacle is the existence of the BBB, which acts as a gatekeeper and prevents most drug molecules from entering the brain, especially large molecular weight agents, such as peptides, monoclonal antibodies, and small-interfering RNA (siRNA) [15–17]. It is worth mentioning that some brain essentials with large molecular weights can cross the BBB by physical transport mechanisms, such as carrier-mediated transport, receptor-mediated transcytosis, and adsorptive-mediated transcytosis pathways. Based on these physiological transport mechanisms, nanotechnology has provided promising strategies for the management of AD drugs or diagnosis, such as liposomes and nanoparticles [18–21]. These nanocarriers loaded with drugs can be further modified with targeting moieties to preferentially bind to putative receptors or transporters expressed at the BBB for enhanced central nervous system (CNS) selectivity and permeability by physiological transport [22–24]. One of the most common and safest nanocarriers for such applications is liposomes [25]. Liposomes are made up of natural or synthetic lipids and characterized as lipid bilayer structures that can encapsulate drug molecules in the aqueous core or lipid bilayers and attach the molecules to the exterior [26]. Massive work has highlighted the great potential of liposomes as drug delivery systems due to their biocompatibility, high flexibility, low toxicity, and stability [27–29]. Interestingly, with some modifications of traditional liposomes, Gobbi et al. [30] prepared anionic liposomes composed of phosphatidic acid (PA) and cardiolipin (CL). PA/CL liposomes are endowed with high Aβ affinity due to the occurrence of multivalent interactions, which makes them promising nanovectors for the treatment of AD [31–34].

Soluble Aβ oligomers, also known as Aβ-derived diffusible ligands (ADDLs), seem to be the most neurotoxic species during AD onset and early progression [35, 36]. According to our previous work, oligomeric Aβs could bind to the fibronectin repeat domain of EphB2 and trigger EphB2 degradation, which resulted in impaired trafficking of NMDA receptors and subsequent inhibition of memory-associated long-term potentiation (LTP) [37]. Hence, we developed neuroprotective Pep63 (VFQVRARTVA), a 10-mer peptide, which could block the interaction of EphB2-ADDLs effectively and reverse impaired memory deficits in early AD mouse models by stereotactic administration into the dorsal hippocampus [38].

In this study, we prepared transferrin-modified and Pep63-loaded liposomes (Tf-Pep63-Lip). PA was incorporated into the lipid membrane of liposomes to enhance the Aβ-binding ability. The surface of the liposome was conjugated with transferrin (Tf) to dramatically enhance CNS delivery of Pep63 across the BBB by transferrin receptor-mediated transcytosis [39, 40]. We conducted in vivo and in vitro experiments to evaluate the characterization, brain distribution, and therapeutic efficiencies of Tf-Pep63-Lip in an early AD mouse model, including the underlying mechanism of Pep63-mediated neuroprotection and PA-mediated Aβ clearance effects. The biosafety of the multifunctional liposomes was also investigated to assess the clinical possibilities for AD treatment.

## Results

### Preparation and characterization of multifunctional liposomes

**Fig. 1:**
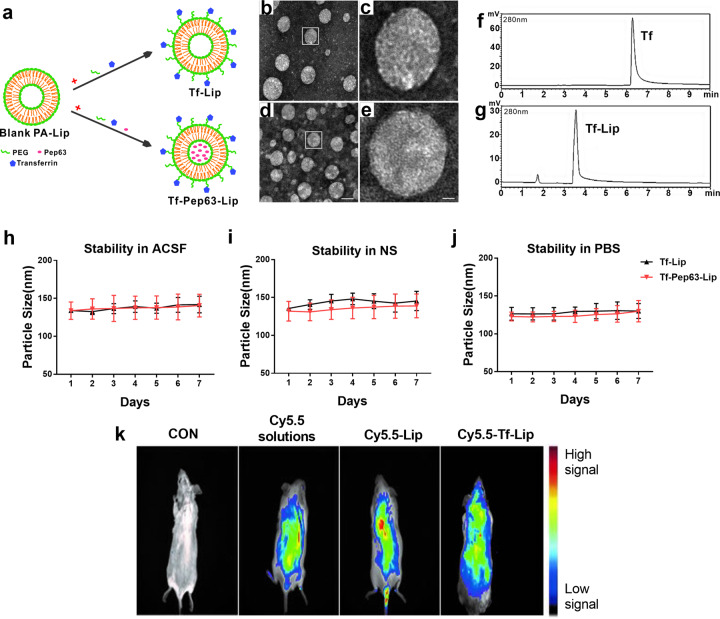
Characterization and biodistribution of the multifunctional liposomes. **a** Schematic diagram of Tf-Lip and Tf-Pep63-Lip preparation. **b**–**e** Morphology and particle size of Tf-Lip and Tf-Pep63-Lip under a transmission electron microscope after negative staining with 2% uranyl acetate solution. **c**, **e** Magnifications of the framed areas of **b** and **d**. Scale bar: to the left panels, 50 nm; to the right panels, 10 nm. **f**, **g** HPLC analysis of the conjugation of Tf to liposomes at 280 nm shows a clear full shift from 6.4 min of non-conjugated Tf (**f**) to 3.6 min of conjugated Tf-Lip (**g**), indicating the successful conjugation of transferrin to liposomes. **h**–**j** Changes in the particle size of Tf-Lip and Tf-Pep63-Lip for seven days in NS (**h**), PBS (**i**), and ACSF (**j**) show stable physical properties in vitro (*n* = 3). **k** Biodistribution of Cy5.5-labeled and transferrin-modified liposomes in living mice. Cy5.5 signals, followed for up to 2 h of post-injection by live animal imaging after intravenous injection of 5 mg lipids/kg mouse of Cy5.5-Lip, Cy5.5-Tf-Lip, and the corresponding dose of Cy5.5 solution, show that transferrin-modified liposomes can cross the brain (*n* = 3). Data are presented as means ± SEM.

Tf-Pep63-Lip and Tf-Lip (Fig. 1a) were prepared according to previously described methods [30, 41]. Fluorescent labeled DSPE-PEG-Cy5.5 was inserted into the membrane of liposomes to obtain Cy5.5-Lip and Cy5.5-Tf-Lip for live imaging of mice. Then, the physical properties of these liposomes were assessed by the average diameter, zeta potential, and PDI (Table 1). The average size of the prepared liposomes was between 124.83 nm and 135.60 nm with a PDI between 0.23 and 0.27, indicating that the liposomes used herein possessed good uniformity. It is widely accepted that liposomes from 100 to 140 nm have certain advantages, such as a longer half-life in blood circulation and avoidance of plasma proteins. Larger liposomes (>250 nm in diameter) can be cleared twice as fast as 100 nm liposomes, while smaller liposomes (<100 nm in diameter) have limited storage capacity. Thus, liposomes prepared in this study possess a longer half-life, higher stability, and encapsulation efficiency [42]. Zeta potential values were relatively negative, suggesting the efficient incorporation of anionic PA into the lipid membrane of liposomes.

**Table 1:** Particle size, zeta potential, and PDI of multifunctional liposomes.

	Particle size (nm)	Zeta potential (mV)	PDI
Tf-Lip	135.60 ± 2.88	−15.40 ± 1.69	0.27
Tf-Pep63-Lip	131.90 ± 22.44	−16.49 ± 0.90	0.24
Cy5.5-Lip	124.83 ± 5.55	−17.26 ± 2.19	0.23
Cy5.5-Tf-Lip	130.17 ± 10.40	−17.49 ± 2.11	0.26

*n* = 3, Data are presented as means ± SEM.

TEM images confirmed that Tf-Lip (Fig. 1b, c) and Tf-Pep63-Lip (Fig. 1d, e) generally presented typical circular shapes with an average diameter of approximately 100 nm. HPLC analysis of Tf and Tf-Lip (Fig. 1f, g) showed a clear full shift from 6.4 min (nonconjugated transferrin) to 3.6 min (DSPE-PEG-Tf), which could indicate the conjugation of transferrin to liposomes by the condensation reaction of DSPE-PEG-COOH with the amino group of transferrin (Fig. S1a). Near-infrared spectrophotometer analysis also confirmed these results (Fig. S1b). Furthermore, the encapsulation efficiency of Pep63 in liposomes quantitated by HPLC was 36.2% (data not shown). The average diameter variations of Tf-Pep63-Lip and Tf-Lip in physical solutions, including ACSF, 0.9% NS, and PBS, were also assessed. The results showed that the sizes of Tf-Lip and Tf-Pep63-Lip did not change in these physical solutions for 7 days (Fig. 1h–j), which indicated stable physical properties so that they could be preserved well. Additionally, the relatively stable properties in physical solutions were the basis of nanocarriers to slow-release the capsuled drugs.

### Brain distribution of Tf-Lip following intravenous administration

The mice were assigned randomly to treat with Cy5.5-Tf-Lip, Cy5.5-Lip, Cy5.5 solution, or NS. Then, biofluorescence images were acquired to monitor the distribution of Cy5.5-Tf-Lip, Cy5.5-Lip, or Cy5.5 solution after intravenous administration (Fig. 1k). The NS-treated mice showed no biofluorescence signals, while mice injected with Cy5.5-labeled liposomes or Cy5.5 solution presented clear signals. Only the Cy5.5-Tf-Lip group displayed Cy5.5 signals in the brains of mice following administration, which suggested that Tf-Lip could bypass the BBB to allow therapeutic drug access to the brain. Additionally, mice were injected with 5 mg lipids/kg rhodamine-labeled and transferrin-modified liposomes (Tf-Rhod-Lip) or Rhod-Lip and then sacrificed for immunofluorescence. Rhodamine signals were detected in the dorsal hippocampal regions of the mice treated with Tf-Rhod-Lip (Fig. S2). Thus, Tf-Lip is a promising carrier for the delivery of Pep63 to the brain by transferrin receptor (TfR)-mediated transcytosis.

### Tf-Pep63-Lip ameliorated memory deficits and decreased Aβ1-42 levels in 6-month-old APP/PS1 mice

**Fig. 2:**
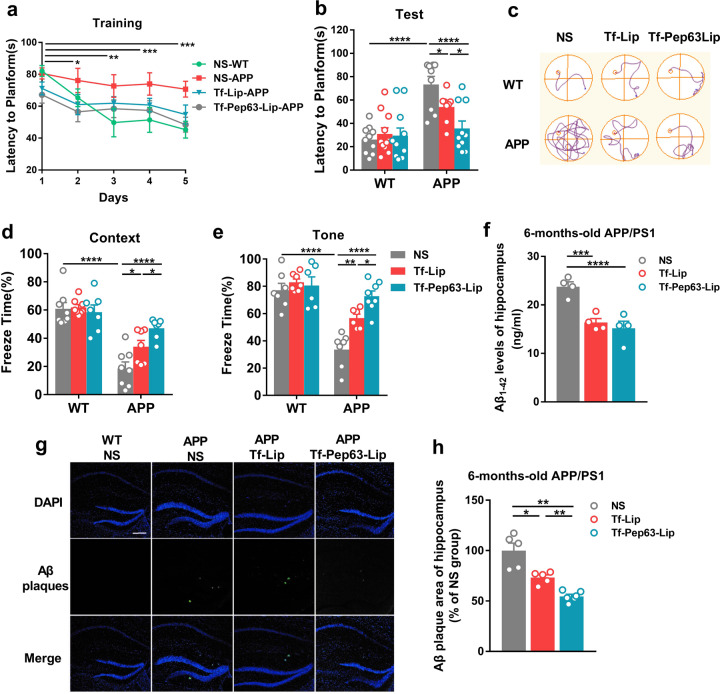
Tf-Pep63-Lip and Tf-Lip rescue memory deficits and decrease Aβ1-42 levels in APP/PS1 mice. Six-month-old APP/PS1 and WT mice were treated with Tf-Lip or Tf-Pep63-Lip at a lipid dose of 5 mg/kg intravenously every other day for 30 days or treated with the same volume of 0.9% NS as negative controls. **a** Escape latency was significantly reduced after training compared with the first day (*n* = 10). **b** APP/PS1 mice injected with Tf-Pep63-Lip and Tf-Lip took less time to reach the platform than NS-treated mice (*n* = 10). **c** Representative swimming path on the testing day. **d**, **e** Tf-Pep63-Lip and Tf-Lip improved impaired context-dependent (**d**) and tone-dependent (**e**) fear memory in APP/PS1 mice compared with NS-treated APP/PS1 mice (*n* = 8). **f** Both Tf-Pep63-Lip and Tf-Lip remarkably decreased Aβ1-42 levels in the hippocampus of APP/PS1 mice, as measured by ELISA (*n* = 4). **g** Representative immunofluorescence images of Aβ1-42 plaques (green) in hippocampal brain sections of APP/PS1 mice treated with 0.9% NS, Tf-Lip, or Tf-Pep63-Lip at a lipid dose of 5 mg/kg intravenously. **h** The area of Aβ1-42 plaques was quantified and normalized to NS-treated APP/PS1 mice. Tf-Lip and Tf-Pep63-Lip significantly decreased the area of Aβ1-42 plaques in the hippocampus of APP/PS1 mice (*n* = 5). Scale bar: 200 µm. *P < 0.05, **P < 0.01, ***P < 0.001, ****P < 0.0001, significantly different. Data are presented as means ± SEM.

To determine the effect of multifunctional liposomes on impaired learning and memory in AD mice, 6-month-old APP/PS1 mice and age-matched wild-type (WT) littermates were intravenously injected with Tf-Pep63-Lip, Tf-Lip or saline as a vehicle every other day for 30 days. Subsequently, spatial learning and memory were assessed by the Morris water maze (MWM), while fear conditioning memory was determined by the fear-conditioning test (FC). For the MWM, the time for mice to locate the escaping platform showed a significant decrease compared with the first day (Fig. 2a), indicating successful training during the trial days. On the test day, APP/PS1 mice showed defects in the acquisition task compared with the vehicle control (Fig. 2b, c). Tf-Pep63-Lip-injected and Tf-Lip-injected, but not vehicle-injected, APP/PS1 mice took less time to find the platform, which indicated that both Tf-Pep63-Lip and Tf-Lip significantly rescued spatial learning and memory under the dosing regimen. Additionally, APP/PS1 mice treated with Tf-Pep63-Lip spent less time reaching the platform than Tf-Lip mice. Similarly, both Tf-Pep63-Lip and Tf-Lip improved both impaired context-dependent and tone-dependent fear memory in APP/PS1 mice. Tf-Pep63-Lip showed more protective effects than Tf-Lip (Fig. 2d, e). Herein, we suggested that Tf-Pep63-Lip and Tf-Lip could ameliorate both hippocampus-dependent and amygdala-dependent memory loss in 6-month-old APP/PS1 mice, and that the protective effects of Pep63 and PA-liposomes were cooperative.

We next examined the total Aβ1-42 levels and Aβ plaques in the AD model mice after administration of Tf-Pep63-Lip and Tf-Lip for 30 days. The total amount of Aβ1-42 in the hippocampus was decreased by Tf-Lip and Tf-Pep63-Lip compared with the saline group (Fig. 2f). Consistently, the deposition of Aβ plaques in the hippocampus (Fig. 2g, h) and cortex (Fig. S3a-b) of Tf-Lip-injected and Tf-Pep63-Lip-injected mice were also decreased. The immunoblotting results further confirmed that Tf-Lip and Tf-Pep63-Lip reduced the level of soluble Aβ oligomers in the hippocampus (Fig. S3c). It is worth-mentioning that APP/PS1 mice injected with Tf-Pep63-Lip showed a smaller Aβ plaque area (Fig. 2g, h) but similar total Aβ1-42 levels (Fig. 2f) to that of Tf-Lip. These results demonstrated that Tf-Lip may accelerate the clearance of Aβ while Pep63 may also slow the accumulation of Aβ.

### Tf-Pep63-Lip accelerated the Aβ chemotaxis and Aβ phagocytosis in microglia

**Fig. 3:**
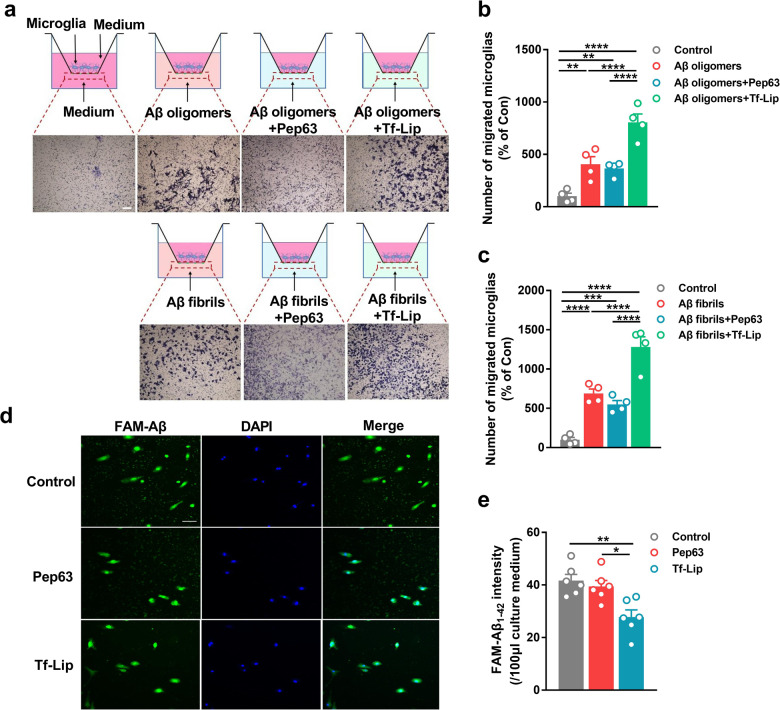
Tf-Lip accelerates the Aβ chemotaxis and Aβ phagocytosis of microglia. **a** Schematic diagram of the Transwell test and representative graphs of primary microglial migration in designed conditions. Scale bar: 200 µm. **b**, **c** Primary microglial migration induced by 100 µM Aβ oligomers (**b**) and fibrils (**c**) in the presence of 2.5 μg/ml Pep63 or 72.5 μg lipids/ml Tf-Lip for 24 h was quantified and normalized to the number of control microglia (*n* = 4). **d** Representative graphs of FAM-Aβ engulfed by primary microglia in the presence of 2.5 μg/ml Pep63 or 72.5 μg lipids/ml Tf-Lip for 30 min. **e** The intensity of FAM-Aβ in the cultured medium after incubation with Pep63 or Tf-Lip for 30 min (*n* = 6). Scale bar: 50 µm. *P < 0.05, **P < 0.01, ***P < 0.001, ****P < 0.0001, significantly different. Data are presented as means ± SEM.

How Tf-Pep63-Lip exert a protective role in early AD mice? In the AD brain, microglia always play a key role in detecting aberrant Aβ species and then engulfing them, especially during the early phase of AD pathogenesis. Hence, the Transwell assay and Aβ phagocytosis assay were conducted to examine the effects of Tf-Lip and Pep63 on the Aβ clearance in microglia. First, we successfully prepared Aβ oligomers and fibrils in vitro (Fig. S4a). Aβ1-42 oligomers and fibrils could significantly induce more primary microglia to migrate from the upper chamber to the lower chamber (Fig. 3a–c). As expected, coincubation with Tf-Lip induced more microglia to migrate toward the lower chamber than incubation with Aβ1-42 oligomers alone (Fig. 3a, b). Consistently, coincubation with Tf-Lip also increased the number of microglia migrating to the lower chamber (Fig. 3a, c) compared with that of Aβ fibrils alone. Pep63 showed no effect on the number of migrated microglia. The results of the Aβ phagocytosis assay indicated that primary microglia could engulf exogenous FAM-Aβ in the culture medium (Fig. 3d). By detecting FAM signals in the upper culture medium, we found that microglia could engulf more FAM-Aβ in the presence of Tf-Lip (Fig. 3e). Additionally, Pep63 had no effect on Aβ phagocytosis, which may explain the similar effect of Tf-Pep63-Lip and Tf-Lip on total Aβ1-42 levels in APP/PS1 mice. These results were consistent with previous reports that anionic liposomes could reduce the Aβ levels in the brains of AD mice [34]. Herein, we demonstrated that anionic PA in Tf-Lip may facilitate Aβ removal by accelerating chemotaxis and phagocytosis of microglia.

### Tf-Pep63-Lip blocked Aβ1-42 aggregation and disaggregated Aβ1-42 assembly in vitro

**Fig. 4:**
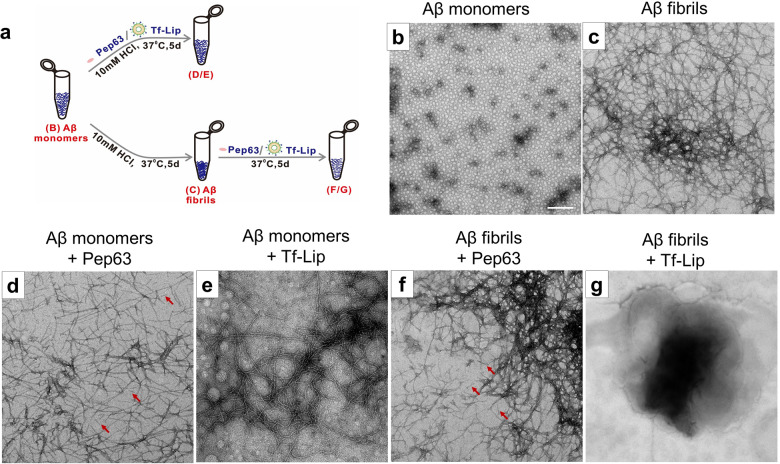
Tf-Lip and Pep63 block the aggregation of Aβ1-42 monomers or disaggregate Aβ1-42 fibrils in vitro. **a** Schematic diagram of the preparation of Aβ aggregates. **b** Electron micrograph of 100 μM Aβ1-42 monomers. **c** Aβ1-42 fibrils of 100 μM Aβ1-42 monomers formed after 5 days incubation with 10 mM HCl at 37 °C. **d**, **e** Final Aβ1-42 assemblies of 100 μM Aβ1-42 monomers formed after 5 days incubation with 2.5 μg/ml Pep63 (**d**) or 72.5 μg lipids/ml Tf-Lip (**e**) at 37 °C for 5 days. **f**, **g** Final Aβ1-42 assemblies of 100 μM Aβ fibrils obtained after incubation with Pep63 (**f**) or Tf-Lip (**g**) at 37 °C for 5 days. Arrows are indications for Pep63. Scale bar: 200 nm.

Previous clues have supported that anionic phospholipid-based liposomes could target Aβ1-42 monomers as well as aggregated forms (Aβ oligomers and fibrils) with high affinity [30, 31]. Pep63 could also competitively bind with Aβ1-42 oligomers. We hypothesized that anionic PA-based Tf-Lip and Pep63 may change the physical properties of Aβ1-42 assemblies by combining with them in vitro. Herein, TEM experiments were conducted to investigate the interaction of Tf-Lip or Pep63 with different forms of Aβ1-42 (Fig. 4a). As shown in Fig. 4b, c, Aβ fibrils were prepared with a higher degree of aggregation than Aβ monomers by incubation of 100 μM Aβ1-42 with 10 mM HCl solutions at 37 °C for 5 days. However, when co-incubation with Pep63 or anionic Tf-Lip in the same condition as Aβ fibrils, Aβ1-42 monomers aggregated into the Aβ fibrils with lower-degree (Fig. 4d, e). The structure of preformed Aβ fibers was significantly disrupted after 5 days of coincubation with anionic Tf-Lip (Fig. 4g) but did not change after coincubation with Pep63 (Fig. 4f). These results were consistent with the immunofluorescence assay, which indicated that both Tf-Lip and Pep63 could reduce Aβ plaques. Tf-Lip reduced the Aβ burden, likely because of the occurrence of anionic PA-mediated blockade of Aβ aggregation, disruption of Aβ fibers and activation of the microglial response. Moreover, Pep63 inhibited Aβ aggregation to decrease Aβ plaques in APP/PS1 mice.

### Tf-Pep63-Lip rescued impaired trafficking of NMDA receptors

According to our previous studies [37, 38], EphB2 colocalizes with NMDA receptors and acts as a key regulator of synaptic localization of NMDA receptors. Aβ oligomers could bind with EphB2 and reduce the surface expression of EphB2 in the hippocampus of 6-month-old APP/PS1 mice. The surface expression of GluN2B-containing NMDA receptors and the tyrosine 1472 site (Y1472, the main phosphorylation site of GluN2B), the basis of LTP, also decreased due to the impaired trafficking induced by dysfunctional EphB2. Neuroprotective Pep63 has been confirmed to disturb the interactions of EphB2-Aβ oligomers and subsequently rescue impaired learning and memory in APP/PS1 mice.

**Fig. 5:**
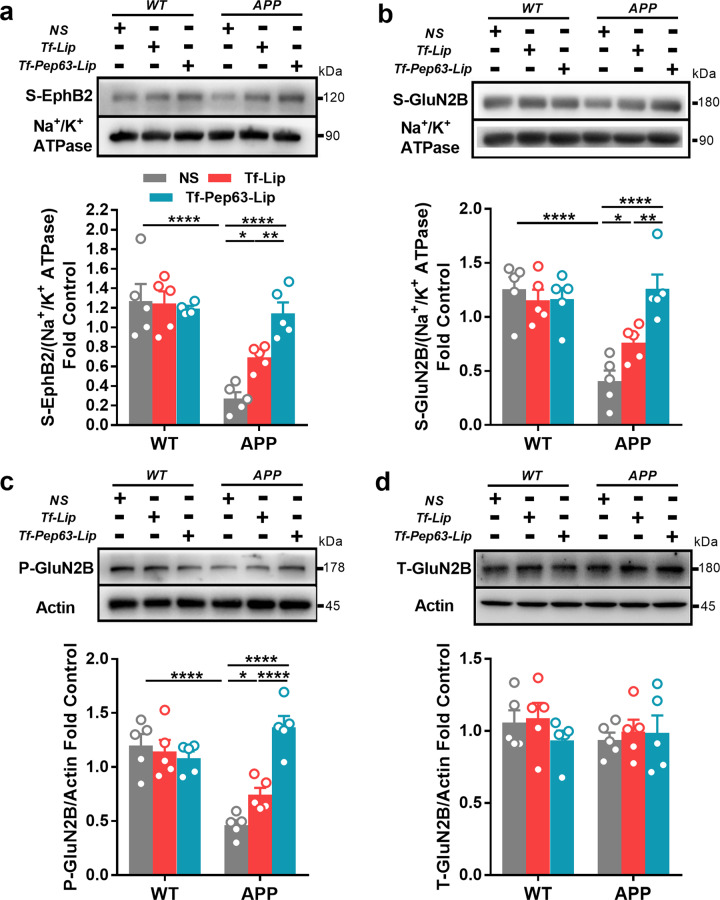
Tf-Pep63-Lip and Tf-Lip rescue the surface expression of EphB2 and GluN2B-containing NMDA receptors in the hippocampus of APP/PS1 transgenic mice. **a** Intravenous administration of Tf-Pep63-Lip and Tf-Lip at a lipid dose of 5 mg/kg for 30 days significantly rescued the reduced surface expression of EphB2 in APP/PS1 transgenic mice (APP) (*n* = 5). **b** Tf-Pep63-Lip and Tf-Lip significantly increased the reduced surface expression of GluN2B subunits in APP/PS1 transgenic mice (*n* = 5). **c** Tf-Pep63-Lip and Tf-Lip remarkably enhanced the phosphorylation levels of GluN2B at pY1472 (p-GluN2B) in APP/PS1 transgenic (*n* = 5). **d** Tf-Pep63-Lip and Tf-Lip did not affect the total expression of GluN2B subunits in APP/PS1 transgenic mice (*n* = 5). *P < 0.05, **P < 0.01, ****P < 0.0001, significantly different. Data are presented as means ± SEM.

To further confirm the therapeutic effects of Tf-Pep63-Lip and Tf-Lip, we first examined surface expressions of EphB2 and GluN2B in the hippocampus of APP/PS1 mice after administration of these agents. As expected, Tf-Pep63-Lip and Tf-Lip increased the surface expressions of EphB2 and GluN2B-containing NMDA receptors in APP/PS1 mice (Fig. 5a, b). In addition, the decreased phosphorylated (pY1472) level of GluN2B was rescued by Tf-Pep63-Lip and Tf-Lip (Fig. 5c). Tf-Pep63-Lip showed more positive effects on rescuing the trafficking of NMDA receptors in APP/PS1 mice than Tf-Lip did (Fig. 5a–c). No change was found in the expression of total GluN2B after treatment, indicating that Tf-Pep63-Lip and Tf-Lip improved synaptic plasticity by rescuing the impaired NMDA trafficking in APP/PS1 mice instead of increasing their expression (Fig. 5d).

### The biosafety of Tf-Pep63-Lip in vivo and in vitro

**Fig. 6:**
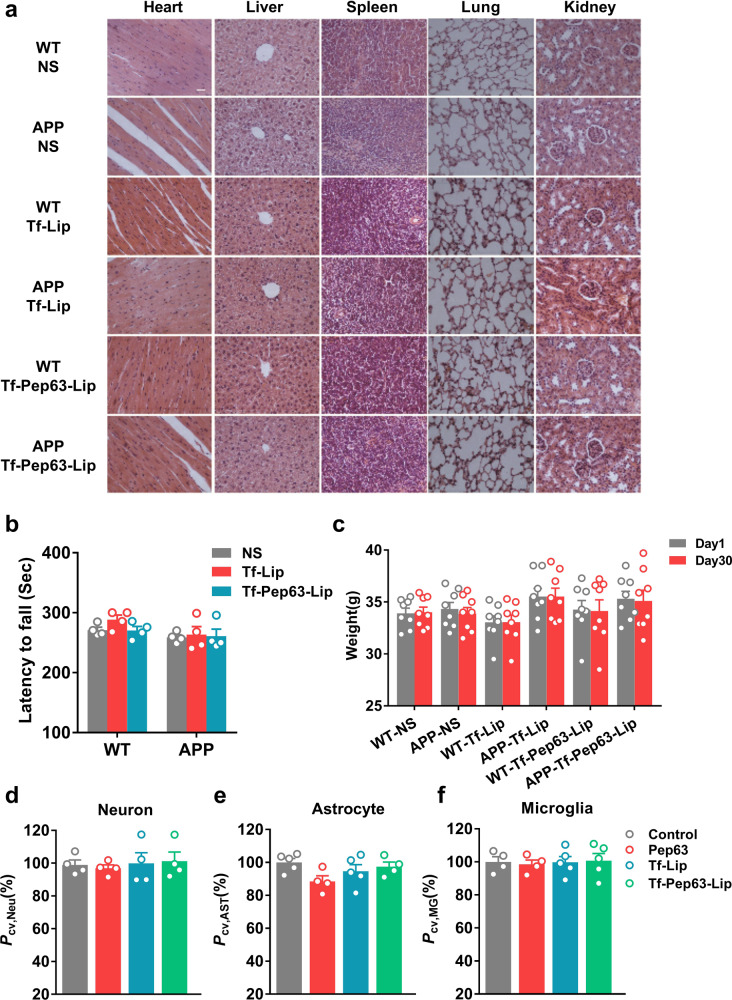
Tf-Pep63-Lip and Tf-Lip do not induce adverse effects in vivo and in vitro. **a** No pathological changes were found in the main organs after 30 days of treatment with Tf-Pep63-Lip, Tf-Lip, or NS in 6-month-old APP/PS1 and WT mice (*n* = 3). **b** Administration of Tf-Pep63-Lip, Tf-Lip, or NS did not affect the motor function of APP/PS1 and WT mice (*n* = 5). **c** The weight of APP/PS1 and WT mice did not change after 30 days of treatment with Tf-Pep63-Lip, Tf-Lip, or NS (*n* = 8). **d**–**f** The viability of hippocampal primary neurons (**d**), astrocytes (**e**), and microglia (**f**) showed no significant change after incubation with 2.5 µg/ml Pep63, lipid doses of 72.5 µg/ml Tf-Lip and Tf-Pep63-Lip for 4 h (*n* = 5). Data are presented as means ± SEM.

The biosafety of Tf-Pep63-Lip and Tf-Lip was also evaluated. HE staining of the organs and CCK-8 assays were conducted to evaluate morphological and cellular alterations, while rotarod tests and weight changes were performed to roughly evaluate motor and metabolic functions. After 30 days’ administration of Tf-Pep63-Lip, Tf-Lip, or saline in APP/PS1 and WT mice, no obvious morphological alterations were detected in the hearts, livers, spleens, lungs, and kidneys (Fig. 6a). In addition, administration of Tf-Pep63-Lip or Tf-Lip had no negative effect on motor activity (Fig. 6b) or the weight of mice (Fig. 6c). Consistently, no toxicity was detected on primary neurons, astrocytes, or microglia via cell viability assays after incubation with these agents, indicating the biosafety of Tf-Pep63-Lip and Tf-Lips in the CNS (Fig. 6d–f and Fig. S4b–d). Taken together, in vivo and in vitro data suggested that Tf-Pep63-Lip and Tf-Lip might be safe under the dosing regimen used in our experiment. This significant biosafety makes Tf-Pep63-Lip great potential for future application in AD therapy.

## Discussion

AD remains one of the biggest global health challenges and has caused an emotional and economic burden to aged people. Unfortunately, there are no effective drugs for this disease so far. Pep63, a novel and neuroprotective peptide, was proven to significantly improve learning and memory deficits in 6-month-old APP/PS1 mice in our previous work. We successfully constructed Tf-Pep63-Lip, the transferrin-modified PA-liposomes loaded with Pep63 in the present research, which could across the BBB, significantly reduce the Aβ burden, rescue the impaired NMDA trafficking and improve cognitive deficits.

We utilized liposomes composed of an additional matrix of POPC, Chol, DSPE-PEG2000-COOH, Tf, and PA for the following reasons. First, POPC and Chol, the commonly used raw materials for the preparation of liposomes, could form a stable lipid bilayer structure and encapsulate with Pep63 in the hydrophilic core. The insertion of cholesterol could not only enhance membrane surface motional behavior but also strengthen vesicle stability [42]. Second, a PEG chain with length of 2 kDa was chosen to hinder the adsorption of blood opsonin and increase the circulating time for liposomes to reach the therapeutic target. Transferrin is a commonly used strategy for liposome delivery to the brain by TfRs, a transmembrane glycoprotein highly expressed on brain endothelial cells [25]. Though multiple lines of evidence indicate that BBB is damaged in neurodegenerative diseases, it should be noted that measurements of TfR levels in microvessels from human postmortem and 3xTg-AD mouse brains show no difference compared with the control group [43], which supports TfRs as a promising vector target for drug delivery into the brain in AD cases. PA, an anionic phospholipid, is inserted into the lipid bilayer of liposomes to endow liposomes with high Aβ affinity for combined therapy.

In this study, we provided evidence that Tf-Pep63-Lip could remarkably reduce Aβ plaques or Aβ fibrils. This is mainly because of the occurrence of PA-Aβ multivalent interactions, which may disturb the hydrophobic and electrostatic interactions between Aβ aggregates and decrease the stability of the overall Aβ fibril structures [44]. In addition, Pep63 could block Aβ fiber formation, likely because it was designed based on the binding sites of the EphB2 FN domain with Aβ1-42. This means that Pep63 shares the same structure with the physiological ligand of Aβ1-42 on neurons and therefore possesses a high affinity for Aβ1-42. Thus, Pep63, as well as anionic PA-based Tf-Lip, could inhibit Aβ1-42 aggregation by combining with Aβ1-42 and then reducing the binding sites between Aβ aggregates. The membrane-anchored Aβ can accelerate Aβ plaque formation and exacerbate the disruption of membranes in AD brain [45–47]. Thus, the bionic membrane of liposomes can also competitively provide raft-like or liquid-ordered domains as the native cellular membrane, where Aβ accumulates. Liposome-anchored Aβ then accelerates Aβ aggregation and exacerbates the disruption of the membrane, followed by the release of Pep63 from liposomes and the reduction of Aβ attachment to the cellular membrane.

We also supported that Tf-Lip could significantly accelerate microglial chemotaxis toward Aβ1-42 and reduce Aβ1-42 levels. Although previous clues have confirmed the effect of PA on Aβ clearance, the underlying mechanism is still unknown. Herein, we provided evidence that microglia mediate PA–Aβ interactions. Interestingly, the triggering receptor expressed on myeloid cells 2 (TREM2), a microglial surface receptor, can sense a wide variety of lipids from the neuronal membrane and work as anionic lipid receptors. The extracellular domain of TREM2 is rich in arginine residues that may form salt bridges with polyanions [48]. Therefore, TREM2 can bind with anionic lipids and then trigger a microglial response. We hypothesized that anionic components of Tf-Lip (PA) may bind Aβ aggregates with high affinity and that the PA-Aβ conjugate could be sensed by the TREM2 receptor through PA-TREM2 interactions, similar to the sandwich effect (PA-Aβ-TREM2), thereby triggering the microglial response for the clearance of Aβ species. Our work confirmed that PA-liposomes accelerated the chemotaxis of microglia and made Aβ more easily be recognized by microglia. Additionally, it’s been proved that anionic liposomes in the periphery can also reduce the Aβ levels in the brain by the so-called “sink effect”, a mechanism that draws excess Aβ out of the brain by enhancing peripheral Aβ clearance [34]. Thus, Tf-Pep63-Lip in the periphery may also play an active role.

Another issue to be considered is the role of fibrillar Aβ in AD pathology, as some studies have shown that fibrillar Aβ triggered microglial dysfunction in AD mouse models [49], while other studies have reported that dense-core Aβ plaques were protective against AD [50]. Our findings supported that Tf-Pep63-Lip could rescue cognitive deficits and Aβ plaques in an AD mouse model. The decreased Aβ plaques were mainly the consequence of clearance of Aβ (PA) and interference of Aβ oligomers (Pep63), which were recognized as most destructive in AD. Tf-Pep63-Lip could also improve NMDA receptors-mediated synaptic plasticity by interfering the interaction between EphB2 and Aβ oligomer. As expected, the PA in Tf-Lip might indirectly rescue the trafficking of NMDA receptors by reducing Aβ levels. Learning and memory tests also confirmed these results in early AD mice.

The remarkable advantage of Tf-Pep63-Lip is the safety of the liposomes, which have been widely used in the clinic [29]. Although recent clinical trials have witnessed the hope of immunotherapy in improving cognitive function in patients, autoimmunity-related adverse effects may be the limitation for future use among elderly individuals who have poor immunity. Therefore, Tf-Pep63-Lip, with limited adverse effects, may be a promising drug for combination therapy with agents targeting other pathologies of AD. It should also be pointed out that more clinical research is required for further therapeutic evaluation of Tf-Pep63-Lip in AD patients due to the complex and changeable pathology of humans. Additionally, AD patients with abnormal iron levels should use the Tf-Pep63-Lip with caution, because transferrin is closely related to iron metabolism.

**Fig. 7:**
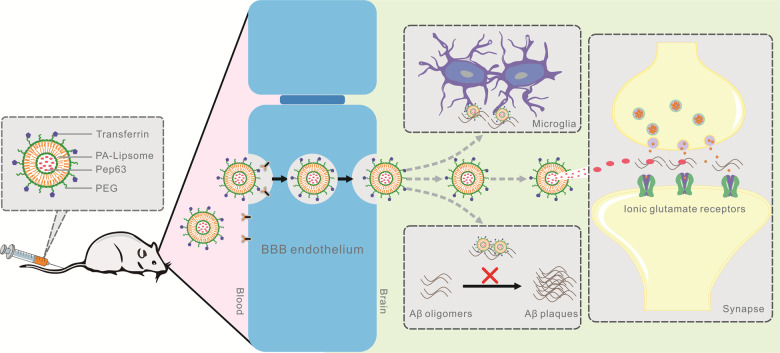
Schematic diagram of transferrin-modified and Pep63-loaded phosphatidic acid (PA)-liposomes (Tf-Pep63-Lip) for combination therapy of AD. After entering the brain by transferrin-mediated transcytosis, the multifunctional liposomes facilitate Aβ chemotaxis and Aβ phagocytosis of microglia, hinder Aβ1-42 aggregation, disaggregate Aβ assemblies, and rescue NMDAR trafficking with joint interference of PA and Pep63. Thus, it may serve as a safe and efficient agent to rescue impaired synaptic plasticity and reduce the Aβ burden in the brain for early AD therapy.

In summary, we successfully constructed transferrin-modified and Pep63-loaded PA-liposomes and found that Tf-Pep63-Lip were effective Aβ-targeting and well-tolerated therapeutic agents for combination therapy in early AD mice. With the collaboration of Pep63, PA, and transferrin, Tf-Pep63-Lip was proven to bypass the BBB into the brain and could rescue cognitive impairment in AD mice. Tf-Pep63-Lip effectively improved synaptic plasticity and accelerated the clearance of Aβ burden in APP/PS1 mice in the following ways (Fig. 7): (1) inhibiting Aβ1-42 aggregation; (2) disaggregating Aβ fibrils; (3) facilitating Aβ chemotaxis/phagocytosis of microglia; and (4) rescuing NMDA receptors trafficking. These findings demonstrated that Tf-Pep63-Lip is a potential agent for AD treatment by intervention with various Aβ forms that correspond with different pathological phases. Nevertheless, it was critical to further explore the therapeutic and adverse effects in clinical trials.

## Materials and methods

### Materials

DSPE-PEG2000-COOH, transferrin (Tf), 1,1,1,3,3,3-hexafluoro-2-propanol (HFIP), dimethyl sulfoxide (DMSO), and poly-d-lysine (PDL) were purchased from Sigma-Aldrich (St. Louis, Missouri, USA). 1-Palmitoyl-2-oleoyl-sn-glycerol-3-phosphocholine (POPC) was obtained from AVT (Shanghai, China). Cholesterol (Chol), N-(3-dimethylaminopropopyl)-N-ethylcarbodiimide hydrochloride (EDC·HCl), N-hydroxysuccinimide (NHS), and acetonitrile were supplied by Aladdin (Shanghai, China). Pep63 (VFQVRARTVA) and Aβ1-42 were synthesized by Bankpeptide (Hefei, China). DSPE-PEG2000-Cy5.5 and dimyristoyl-phosphatidic acid (PA) were purchased from Ruixi (Xian, China). Acetonitrile was from TEDIA (Ohio, USA). Trypsin-EDTA, NeuroBasal medium, B27, GlutaMax, and DMEM were purchased from Invitrogen (California, USA). Fetal bovine serum (FBS) was obtained from Biochrom AG (Berlin, Germany). Other chemical reagents were supplied by SINOPHARM (Beijing, China).

### Animals

Six-month-old APP/PS1 transgenic mice overexpressing amyloid precursor protein (APP 695swe) and presenilin 1 (PS1-dE9) and WT mice (nontransgenic littermates of APP/PS1 mice) were purchased from the Model Animal Research Center of Nanjing University. Sprague-Dawley rats (SD) were obtained from the animal center of Xuzhou Medical University. Animals were housed under a barrier environment with standard temperature (22 ± 2 °C) and humidity (40-50%), as well as a dark/light cycle of 12 h (8:00 A.M.–8:00 P.M.). All animals have free access to food and water. All operations were approved by the Institutional Animal Care and Use Committee (IACUC) of Xuzhou Medical University in compliance with National Institutes of Health standards. All animals were allocated to experimental groups randomly.

### Cells

Primary neurons, astrocytes, and microglia were generated as previously described [51]. Briefly, the hippocampus from 19 to 21 embryonic SD rats was isolated and dissociated with trypsin-EDTA (0.25%). For primary neuron culture, cell suspensions were transferred to 96-well plates coated with PDL (0.05 mg/ml) and maintained in the presence of NeuroBasal medium with B27 and GlutaMax. After 18–21 days of culture, neurons were used for in vitro experiments. For microglia and astrocytes, cell suspensions were cultured in T-75 flasks with DMEM supplemented with 10% FBS until mixed glial cultures were completely confluent. Microglia was shaken off the mixed brain glial cell cultures on an orbital shaker for 6 h at 220 cycles per minute, and then seeded onto PDL-pretreated 96-well plates. The remaining astrocytes were harvested by 0.25% trypsin-EDTA solution and cultured with 10% FBS/DMEM. Experiments of astrocytes were conducted after 3–5 generations for further purification. All cells were kept at 37 °C in a humidified incubator with 5% CO_2_/95% air, and the culture medium was changed every 2–3 days. All primary cells were allocated to experimental groups randomly.

### Preparation and characterization of liposomes

Preparation of multifunctional liposomes was carried out as previously described by the thin-film hydration method with some modifications [30]. The blank PA-liposomes were composed of a matrix of POPC, Chol, DSPE-PEG2000-COOH and PA (60:20:6:3, molar ratio). The lipids above were resuspended in chloroform/methanol (2:1, v:v) and dried under a gentle stream by a vacuum pump to remove the organic solvent. The lipid film was hydrated with saline or 2.5 μg/ml pep63 in saline and then sonicated in a water bath ultrasound (40 °C) for 10 min and an ultrasonic (200 W, 3 s sonication, 2 s interval) for 10 min separately. The mixed solutions were transferred into a dialysis bag (MWCO: 3000 Da) and stirred in saline for 12 h to remove the free lipids or drugs. The liposomes were activated by EDC·HCl and NHS at pH 5.5 (DSPE-PEG2000-COOH:EDC·HCl:NHS = 1:40:100, molar ratio), the pH of the liquid was tuned to 7.5, and Tf was added. Tf-Pep63-Lip was isolated from unbound Tf or Pep63 by a Sephadex G100 column with saline as the eluent.

The average diameter, zeta potential, and polydispersity index (PDI) were measured by a Nicomp 380 ZLS dynamic laser light scattering (DLLS) instrument (PSS, Florida, USA). Multifunctional liposomes (*n* = 3) were resuspended in saline (NS, 1:14, v-v), phosphate-buffered saline (PBS, 1:14, v-v) or artificial cerebral-spinal fluid (ACSF, containing 126 mM NaCl, 2.5 mM KCl, 1 mM MgCl_2_, 1 mM CaCl_2_, 1.25 mM KH_2_PO_4_, 26 mM NaHCO_3_, and 20 mM glucose, 1:388, v-v) to mimic the condition in biological fluids and examine the stability for 7 days. The variations of the average diameter in these suspensions were determined every day by DLLS.

### High-performance liquid chromatography (HPLC)

The encapsulation efficiency (EE) of Pep63-loaded liposomes was measured by LC-20AT HPLC (Shimadzu, Kyoto, Japan). Pep63 powder was precisely weighed and prepared into 5, 10, 20, 50, 100, and 200 μg/ml Pep63 solutions. Pep63-loaded liposomes and Pep63 solutions were dissolved in methanol, and then the supernatant was collected for HPLC analyses. The concentration standard curve of Pep63 was determined by the peak areas of Pep63 at different concentrations. The content of Pep63 encapsulated in liposomes was measured according to the peak area and concentration standard curve of Pep63. HPLC was conducted as follows: Agilent TC-C18 column (5 µm, 4.6 × 250 mm), mobile phase: 0.1% trifluoroacetic in aqueous solution and acetonitrile (40:60, v/v), flow rate: 1 ml/min, injection volume: 20 μl, detection wavelength: 280 nm, column temperature: 25 °C. Encapsulation efficiency was estimated as EE = (Pep63 content encapsulated in liposomes/total Pep63 content) × 100%. The assessment of the conjugation reaction of Tf and liposomes was also analyzed by HPLC at 280 nm as described above.

### Live animal imaging system

Cy5.5-labeled Tf-Lip (Cy5.5-Tf-Lip) and Cy5.5-Lip were prepared as mentioned above (POPC:Chol:DSPE-PEG2000-COOH:DSPE-PEG-Cy5.5:PA = 60:20:3:3:3, molar ratio). The mice (*n* = 3) were injected with Cy5.5-Tf-Lip, Cy5.5-Lip, or Cy5.5 solutions by tail vein injection at a dose of 5 mg lipids/kg. Two hours later, the mice were anesthetized with tribromoethanol (0.24 mg/g, *i.p*.) and lied in a NightOWL II LB 983 imaging system (Berthold Technologies, Bad Wildbad, Germany). Biofluorescence images were acquired by excitation/emission wavelengths to detect Cy5.5 signals (excitation: 630 nm; emission: 700 nm). Images were analyzed by Indigo software.

### Morris water maze

As previously described [38], the experiment was conducted in a circular pool (1.2 m in diameter) filled with white water. A platform was hidden 2 cm below the opaque water. During the first 5 days, mice (*n* = 10) were trained to swim for 90 s, with four trials each day, to find the platform and stay in the position for the 30 s’ location. If animals failed to find the platform, the experimenter gently guided them. On the sixth day, the latency time for mice to reach the platform was recorded to assess spatial memory. The swimming paths and escape latencies were tracked by an Anymaze system during the training and testing phases. The investigator was blinded to the group allocation of animals during the test.

### Fear conditioning

As previously described [52], mice (*n* = 8) were kept in a square box for 3 min to remember the context and then stimulated by a 30’s tone (10 kHz; 75 dB) followed by a foot shock (2 s; 0.7 mA) during the training day. On the next day, mice were sent to the same contextual box without tone stimulations (3 min) or to an unfamiliar contextual box (30 s) with tone stimulations (30 s) to test the contextual and tone-dependent fear conditioning memory, respectively. The movement of mice was detected by an automated infrared system and analyzed with Video Freeze software. The freezing time was recorded when the motor threshold of the mice was under 18. The investigator was blinded to the group allocation of animals during the test.

### Immunofluorescence

Anesthetized mice were perfused with 0.9% saline and 4% paraformaldehyde (PFA) from the left ventricle. Next, the brain was removed and fixed with 4% PFA overnight followed by 30% sucrose for 3–5 days. Serial coronal sections of the hippocampus (30 μm thickness) were acquired using a cryostat (Leica, Wetzlar, Germany). After rinsing with PBS, slices (*n* = 5) were incubated at 4 °C overnight with amyloid Aβ1-42 primary antibody (1:400, Cell Signaling Technology Massachusetts, USA, 14974S) to label Aβ plaques. Brain slices were subsequently incubated for 2 h at room temperature with Alexa 488-conjugated secondary antibodies (1:400, Invitrogen, California, USA, A-11029) and counterstained with DAPI Fluoromount-G (SouthernBiotech, Alabama, USA) for 15 min. Images were acquired by an LSM880 confocal laser (Zeiss, Oberkochen, Germany) and analyzed with ImageJ software.

### ELISA

Fresh hippocampal tissues (*n* = 4) from anesthetized mice were treated with 1% SDS and protease inhibitor. The levels of Aβ in the hippocampus were determined by a sandwich ELISA kit (LanpaiBIO, Shanghai, China) according to the manufacturer’s instructions.

### Preparation of Aβ oligomers and fibrils

Synthetic Aβ1-42 peptides were suspended in HFIP for evaporation and drying to form Aβ1-42 peptide films. Preparation for Aβ oligomers and fibrils was conducted as described previously [53, 54]. Briefly, Aβ1-42 was diluted in DMSO to 5 mM before use. Aβ1-42 oligomers were prepared by diluting 5 mM Aβ1-42 to a final concentration of 100 μM with DMEM and incubating at 4 °C for 24 h. For Aβ1-42 fibrils, 5 mM Aβ1-42 was diluted to 100 μM in 10 mM HCl solutions and incubated at 37 °C for 5 days.

### Transmission electron microscope (TEM) image

To verify the shape and structure of liposomes and Aβ aggregations, 10 μl suspensions of these solutions were dropped onto a carbon-coated 200-mesh copper grid for 5 min and stained with uranyl acetate for 1 min. The excess solution was removed via filter paper and allowed to air dry. To examine the abilities of liposomes to hinder the formation of Aβ fibrils or disaggregate the preformed Aβ fibrils in vitro, 100 μM Aβ1-42 monomers or fibrils were incubated with 2.5 μg/ml Pep63 or 72.5 μg lipids/ml Tf-Lip for 5 days at 37 °C. Then, 10 μl mixed suspensions were negatively stained and investigated by TEM. The images were acquired by a Tecnai G2 Spirit transmission electron microscope (FEI, Oregon, USA)

### Transwell migration assay

With 8.0 µm-pore-size inserts (Millipore, Massachusetts, USA), the primary microglia can migrate from the upper chambers to the lower chambers through the micropore. To assess the effect of Tf-Lip and Pep63 on microglial migration, microglial suspensions (*n* = 4) were transferred to the upper chambers, while 72.5 μg lipids/ml Tf-Lip and 2.5 μg/ml Pep63 were added to the lower chambers with or without Aβ oligomers or Aβ fibrils. After 24 h of incubation at 37 °C in a humidified incubator with 5% CO_2_, microglia on the membrane of the Transwell inserts were fixed with 4% paraformaldehyde and stained with 1% crystal violet for 30 min at room temperature. Then, the un-migrated microglia on the membrane of the upper inserts were carefully removed with humidified and clean cotton. Images were acquired by phase-contrast microscopy (Olympus, Shinjuku, Japan) and Image Analysis software.

### Aβ phagocytosis assay

Primary microglia (*n* = 6) were incubated with 200 nM FAM-Aβ (Anaspec, California, USA) for 30 min as previously described [55]. Then, the mean intensity of FAM-Aβ (excitation: 494 nm; emission: 521 nm) in the upper culture medium was detected by a Varioskan LUX microplate reader (Thermo Scientific, Massachusetts, USA). After removal of the culture medium, the microglia were washed with PBS and fixed with 4% PFA for 30 min. After rinsing with PBS, the cells were counterstained with DAPI Fluoromount-G (SouthernBiotech, Alabama, USA) for 15 min. Images were acquired by an LSM880 confocal laser (Zeiss, Oberkochen, Germany).

### Immunoblotting

After anesthetizing with 2% isoflurane (RWD, Shenzhen, China), mice (*n* = 5) were sacrificed, and the hippocampus was separated. The tissues were immediately frozen in liquid nitrogen and stored at −80 °C or suspended in RIPA lysis buffer containing protease inhibitors (Beyotime, Shanghai, China). The supernatant of the sample was acquired after sonicating for the detection of protein concentration. For membrane proteins, subcellular fractions were separated according to the manufacturer’s instructions for the ProteoExtract kit (Calbiochem, Darmstadt, Germany). Lysates containing an equal amount of protein (5–10 mg) were separated by 7.5% or 10% SDS polyacrylamide gel electrophoresis (SDS-PAGE). Then, proteins were transferred to a PVDF membrane (Merck Millipore, Massachusetts, USA) and blocked with 5% skim milk in Tris-buffered saline containing 0.1% Tween-20 (TBST) for 2 h at room temperature. The membranes were incubated overnight at 4 °C with the following primary antibodies: EphB2 (1:2000; Santa Cruz Biotechnology, Texas, USA, sc-130068), GluN2B (NMDAε2 primary antibody, 1:2000; Santa Cruz Biotechnology, Texas, USA, sc-365597), p-GluN2B (Y1472) (1:2000; Abcam, Cambridge, UK, ab3856), β-actin (1:2000; Santa Cruz Biotechnology, Texas, USA, sc-8432), and Na^+^/K^+^ ATPase (1:2000; Abbkine, California, USA, ABP51894). After rinsing with TBST, membranes were incubated with horseradish peroxidase-conjugated secondary antibodies (1:2000; Beyotime, Shanghai, China) for 2 h at room temperature. Immunoreactive protein bands were detected with chemiluminescent (ECL) substrate (Beyotime, Shanghai, China, A0208/ A0216) and an Alliance Q9 system (UVI Tec, Cambridge, UK). The results were quantified and analyzed with ImageJ software.

### Paraffin sections and HE stain

After mice (*n* = 3) were anesthetized and sacrificed, the hearts, liver, spleen, lungs, and kidneys were quickly harvested and fixed in 10% formalin at room temperature for 2–3 days. Then, fixed organs were embedded in paraffin and cut into serial coronal sections at 4 μm. Subsequently, the slices were stained with hematoxylin and eosin following standard instructions. Images were acquired by a brightfield microscope (Nikon, Tokyo, Japan).

### Rotarod test

Mice (*n* = 4) were trained to hang on the rotarod apparatus (3 cm diameter) with a speed of 10 rpm (10 min) on the first 3 days. During the test phase, the rod mode was changed to a constant acceleration rate of 4-40 rpm. The mean time of two trials (more than 60 min intervals) of mice staying on the rod was recorded in 300 s. The investigator was blinded to the group allocation of animals during the test.

### Cytotoxicity assay

After incubating with 2.5 μg/ml Pep63, 72.5 μg/ml Tf-Lip, and 72.5 μg/ml Tf-Pep63-Lip solutions for 4 h, the culture media of neurons, astrocytes and microglia (*n* = 5) were discarded and replaced with fresh DMEM containing 10% CCK-8 solution (Dojindo, Kumamoto, Japan). Absorbance at 450 nm was measured with a microplate reader 2 h later (Thermo Fisher, Massachusetts, USA). Percentage of cell viability (pcv) = (absorbance of experimental wells − absorbance of blank wells)/(absorbance of control wells − absorbance of blank wells).

### Statistics

The sample size was estimated from preliminary experiments or from reports in the literature. Water maze data in the training phase and stability experiments of liposomes were analyzed by repeated-measures ANOVA followed by post hoc Student–Newman–Keuls (SNK) multiple comparisons (when the *P*-value of Mauchly’s test of sphericity was less than 0.05, Greenhouse-Geisser was applied to correct the degree of freedom). Other data were analyzed via one-way ANOVA followed by the SNK, least-significant difference (LSD, equal variances assumed), or Dunnett’s T3 (equal variances not assumed) for multiple comparisons. Differences were considered significant at *P* < 0.05.

## Supplementary information

Supporting Information

## Data Availability

The data sets used and analyzed during the current study are available from the corresponding author on reasonable request.

## References

[1] Blenkinsop A, van der Flier WM, Wolk D, Lehmann M, Howard R, Frost C (2020). Non-memory cognitive symptom development in Alzheimer’s disease. Eur J Neurol.

[2] Lanctôt KL, Amatniek J, Ancoli-Israel S, Arnold SE, Ballard C, Cohen-Mansfield J (2017). Neuropsychiatric signs and symptoms of Alzheimer’s disease: new treatment paradigms. Alzheimers Dement.

[3] Shampo MA, Kyle RA, Steensma DP (2013). Alois Alzheimer-Alzheimer disease. Mayo Clin Proc.

[4] Farlow MR, Miller ML, Pejovic V (2008). Treatment options in Alzheimer’s disease: maximizing benefit, managing expectations. Dement Geriatr Cogn Disord.

[5] Joe E, Ringman JM (2019). Cognitive symptoms of Alzheimer’s disease: clinical management and prevention. BMJ.

[6] Schneider L (2020). A resurrection of aducanumab for Alzheimer’s disease. Lancet Neurol.

[7] Selkoe DJ (2019). Alzheimer disease and aducanumab: adjusting our approach. Nat Rev Neurol.

[8] Howard R, Liu KY (2020). Questions EMERGE as Biogen claims aducanumab turnaround. Nat Rev Neurol.

[9] Hardy JA, Higgins GA (1992). Alzheimer’s disease: the amyloid cascade hypothesis. Science.

[10] McAllister BB, Lacoursiere SG, Sutherland RJ, Mohajerani MH (2020). Intracerebral seeding of amyloid-β and tau pathology in mice: factors underlying prion-like spreading and comparisons with α-synuclein. Neurosci Biobehav Rev.

[11] Cuello AC (2017). Early and late CNS inflammation in Alzheimer’s disease: two extremes of a continuum. Trends Pharm Sci.

[12] Butterfield DA, Halliwell B (2019). Oxidative stress, dysfunctional glucose metabolism and Alzheimer disease. Nat Rev Neurosci.

[13] Area-Gomez E, Guardia-Laguarta C, Schon EA, Przedborski S (2019). Mitochondria, OxPhos, and neurodegeneration: cells are not just running out of gas. J Clin Invest.

[14] Huang Y, Mucke L (2012). Alzheimer mechanisms and therapeutic strategies. Cell.

[15] Pandit R, Chen L, Götz J (2020). The blood-brain barrier: physiology and strategies for drug delivery. Adv Drug Deliv Rev.

[16] Pardridge WM (2005). The blood-brain barrier: bottleneck in brain drug development. NeuroRx.

[17] Banks WA (2012). Drug delivery to the brain in Alzheimer’s disease: consideration of the blood-brain barrier. Adv Drug Deliv Rev.

[18] Formicola B, Cox A, Dal Magro R, Masserini M, Re F (2019). Nanomedicine for the treatment of Alzheimer’s disease. J Biomed Nanotechnol.

[19] Furtado D, Björnmalm M, Ayton S, Bush AI, Kempe K, Caruso F (2018). Overcoming the blood-brain barrier: the role of nanomaterials in treating neurological diseases. Adv Mater.

[20] Srivastava AK, Roy Choudhury S, Karmakar S (2020). Near-infrared responsive dopamine/melatonin-derived nanocomposites abrogating in situ amyloid β nucleation, propagation, and ameliorate neuronal functions. ACS Appl Mater Interfaces.

[21] Gao H, Liu M, Zhao Z, Yang C, Zhu L, Cai Y (2020). Diagnosis of mild cognitive impairment and Alzheimer’s disease by the plasma and serum amyloid-beta 42 assay through highly sensitive peptoid nanosheet sensor. ACS Appl Mater Interfaces.

[22] Wong HL, Wu XY, Bendayan R (2012). Nanotechnological advances for the delivery of CNS therapeutics. Adv Drug Deliv Rev.

[23] Agrawal M, Saraf S, Saraf S, Dubey SK, Puri A, Patel RJ (2020). Recent strategies and advances in the fabrication of nano lipid carriers and their application towards brain targeting. J Control Release.

[24] Mulvihill JJ, Cunnane EM, Ross AM, Duskey JT, Tosi G, Grabrucker AM (2020). Drug delivery across the blood-brain barrier: recent advances in the use of nanocarriers. Nanomedicine.

[25] Agrawal M, Ajazuddin, Tripathi DK, Saraf S, Saraf S, Antimisiaris SG (2017). Recent advancements in liposomes targeting strategies to cross blood-brain barrier (BBB) for the treatment of Alzheimer’s disease. J Control Release.

[26] Noble GT, Stefanick JF, Ashley JD, Kiziltepe T, Bilgicer B (2014). Ligand-targeted liposome design: challenges and fundamental considerations. Trends Biotechnol.

[27] Michanetzis GP, Markoutsa E, Mourtas S, Missirlis YF, Antimisiaris SG (2019). Hemocompatibility of amyloid and/or brain targeted liposomes. Future Med Chem.

[28] Ross C, Taylor M, Fullwood N, Allsop D (2018). Liposome delivery systems for the treatment of Alzheimer’s disease. Int J Nanomed.

[29] Kapoor M, Lee SL (2017). Liposomal drug product development and quality: current US experience and perspective. AAPS J.

[30] Gobbi M, Re F, Canovi M, Beeg M, Gregori M, Sesana S (2010). Lipid-based nanoparticles with high binding affinity for amyloid-beta1-42 peptide. Biomaterials.

[31] Mancini S, Balducci C, Micotti E, Tolomeo D, Forloni G, Masserini M (2017). Multifunctional liposomes delay phenotype progression and prevent memory impairment in a presymptomatic stage mouse model of Alzheimer disease. J Control Release.

[32] Bana L, Minniti S, Salvati E, Sesana S, Zambelli V, Cagnotto A (2014). Liposomes bi-functionalized with phosphatidic acid and an ApoE-derived peptide affect Aβ aggregation features and cross the blood-brain-barrier: implications for therapy of Alzheimer disease. Nanomedicine.

[33] Balducci C, Mancini S, Minniti S, La Vitola P, Zotti M, Sancini G (2014). Multifunctional liposomes reduce brain β-amyloid burden and ameliorate memory impairment in Alzheimer’s disease mouse models. J Neurosci.

[34] Ordóñez-Gutiérrez L, Re F, Bereczki E, Ioja E, Gregori M, Andersen AJ (2015). Repeated intraperitoneal injections of liposomes containing phosphatidic acid and cardiolipin reduce amyloid-β levels in APP/PS1 transgenic mice. Nanomedicine.

[35] Krafft GA, Klein WL (2010). ADDLs and the signaling web that leads to Alzheimer’s disease. Neuropharmacology.

[36] Benilova I, Karran E, De Strooper B (2012). The toxic Aβ oligomer and Alzheimer’s disease: an emperor in need of clothes. Nat Neurosci.

[37] Hu R, Wei P, Jin L, Zheng T, Chen WY, Liu XY (2017). Overexpression of EphB2 in hippocampus rescues impaired NMDA receptors trafficking and cognitive dysfunction in Alzheimer model. Cell Death Dis.

[38] Shi XD, Sun K, Hu R, Liu XY, Hu QM, Sun XY (2016). Blocking the interaction between EphB2 and ADDLs by a small peptide rescues impaired synaptic plasticity and memory deficits in a mouse model of Alzheimer’s disease. J. Neurosci.

[39] Zuchero YJ, Chen X, Bien-Ly N, Bumbaca D, Tong RK, Gao X (2016). Discovery of novel blood-brain barrier targets to enhance brain uptake of therapeutic antibodies. Neuron.

[40] Luo M, Lewik G, Ratcliffe JC, Choi CHJ, Mäkilä E, Tong WY (2019). Systematic evaluation of transferrin-modified porous silicon nanoparticles for targeted delivery of doxorubicin to glioblastoma. ACS Appl Mater. Interfaces.

[41] Chen ZL, Huang M, Wang XR, Fu J, Han M, Shen YQ (2016). Transferrin-modified liposome promotes α-mangostin to penetrate the blood-brain barrier. Nanomedicine.

[42] Hsieh CJ, Chen YW, Hwang DW (2013). Effects of cholesterol on membrane molecular dynamics studied by fast field cycling NMR relaxometry. Phys Chem Chem Phys.

[43] Bourassa P, Alata W, Tremblay C, Paris-Robidas S, Calon F (2019). Transferrin receptor-mediated uptake at the blood-brain barrier is not impaired by Alzheimer’s disease neuropathology. Mol Pharm.

[44] Takahashi T, Mihara H (2008). Peptide and protein mimetics inhibiting amyloid beta-peptide aggregation. Acc Chem Res.

[45] Jeffrey M, McGovern G, Barron R, Baumann F (2015). Membrane pathology and microglial activation of mice expressing membrane anchored or membrane released forms of Aβ and mutated human Alzheimer’s precursor protein (APP). Neuropathol Appl Neurobiol.

[46] Nagarathinam A, Höflinger P, Bühler A, Schäfer C, McGovern G, Jeffrey M (2013). Membrane-anchored Aβ accelerates amyloid formation and exacerbates amyloid-associated toxicity in mice. J Neurosci.

[47] Michikawa M, Gong JS, Fan QW, Sawamura N, Yanagisawa K (2001). A novel action of alzheimer’s amyloid beta-protein (Abeta): oligomeric Abeta promotes lipid release. J Neurosci.

[48] Wang Y, Cella M, Mallinson K, Ulrich JD, Young KL, Robinette ML (2015). TREM2 lipid sensing sustains the microglial response in an Alzheimer’s disease model. Cell.

[49] Sebastian Monasor L, Müller SA, Colombo AV, Tanrioever G, König J, Roth S (2020). Fibrillar Aβ triggers microglial proteome alterations and dysfunction in Alzheimer mouse models. eLife.

[50] Huang Y, Happonen KE, Burrola PG, O’Connor C, Hah N, Huang L (2021). Microglia use TAM receptors to detect and engulf amyloid β plaques. Nat Immunol.

[51] Skaper SD, Facci L (2018). Culture of neonatal rodent microglia, astrocytes, and oligodendrocytes from the cortex, spinal cord, and cerebellum. Methods Mol Biol.

[52] Gao C, Frausto SF, Guedea AL, Tronson NC, Jovasevic V, Leaderbrand K (2011). IQGAP1 regulates NR2A signaling, spine density, and cognitive processes. J. Neurosci.

[53] Rönicke R, Mikhaylova M, Rönicke S, Meinhardt J, Schröder UH, Fändrich M (2011). Early neuronal dysfunction by amyloid β oligomers depends on activation of NR2B-containing NMDA receptors. Neurobiol. Aging.

[54] Stine WB, Dahlgren KN, Krafft GA, LaDu MJ (2003). In vitro characterization of conditions for amyloid-beta peptide oligomerization and fibrillogenesis. J Biol Chem.

[55] Zhao Y, Wu X, Li X, Jiang LL, Gui X, Liu Y (2018). TREM2 is a receptor for β-amyloid that mediates microglial function. Neuron.

