# Supplementary material for: Transferrin-Pep63-liposomes accelerate the clearance of Aβ and rescue impaired synaptic plasticity in early Alzheimer’s disease models
Source: Cell Death Discov. 2021 Sep 21;7:256. doi: 10.1038/s41420-021-00639-1 (PMC8455582; doi:10.1038/s41420-021-00639-1)
Supplement: Supplementary file 1 — Supporting Information [file 41420_2021_639_MOESM1_ESM.docx]

**Supplementary Information**

**Transferrin-Pep63-Liposomes Accelerate the Clearance of Aβ and Rescue Impaired Synaptic Plasticity in Early Alzheimer’s Disease Mouse Models**

Xiu Yang^1*^, Xu Li^1*^, Le Liu^1*^, Yuanhao Chen^1^, Yue You^1^, Yin Gao^1^, Yueying Liu^1^, Li Yang^1^, Kun Tong^1^, Dishi Chen^1^, Jingru Hao^1^, Nan Sun^1^, Ziming Zhao^2^, and Can Gao^1^

^1^NMPA Key Laboratory for Research and Evaluation of Narcotic and Psychotropic Drugs, Jiangsu Province Key Laboratory of Anesthesiology, Jiangsu Province Key Laboratory of Anesthesia and Analgesia Application, Xuzhou Medical University, Xuzhou, Jiangsu, China; ^2^ Jiangsu Province Key Laboratory of New Drug Research and Clinical Pharmacy, Xuzhou Medical University, Xuzhou, Jiangsu, China

Correspondence: Can Gao ([gaocan@xzhmu.edu.cn](mailto:gaocan@xzhmu.edu.cn)) and Zi-ming Zhao (zmzhao@xzhmu.edu.cn)

209 Tongshan Road, Xuzhou, Jiangsu, 221004

Tel 86-83262301

*Xiu Yang, Xu Li, and Le Liu contributed equally to this work.


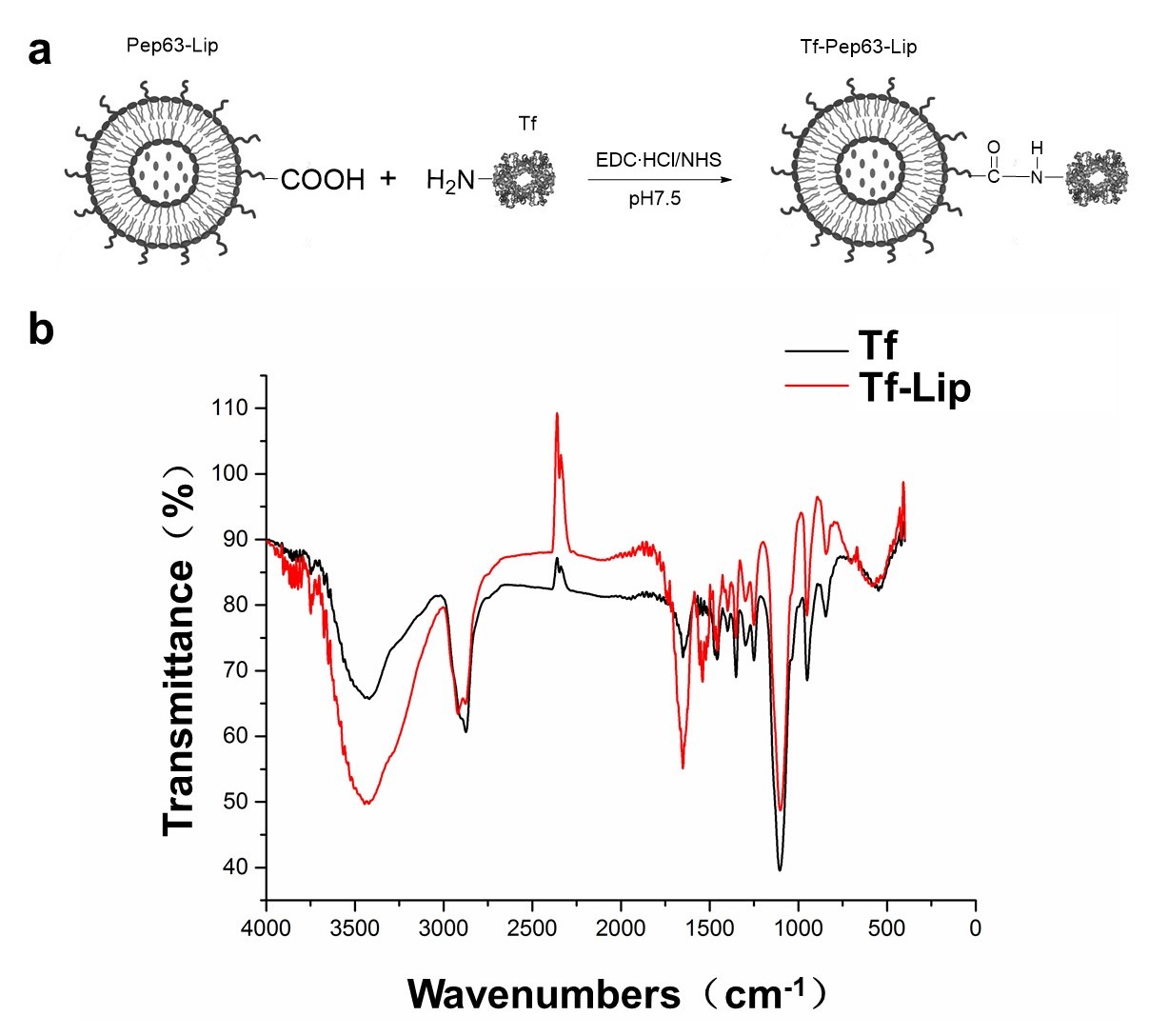


**Figure S1 Transferrin is conjugated to liposomes by condensation reaction.** **a** Schematic diagram of condensation reaction. **b** Near-Infrared Spectrophotometers analysis of transmittance at 2600 cm^-1^ shows a clear shift between Tf and conjugated Tf-Lip.


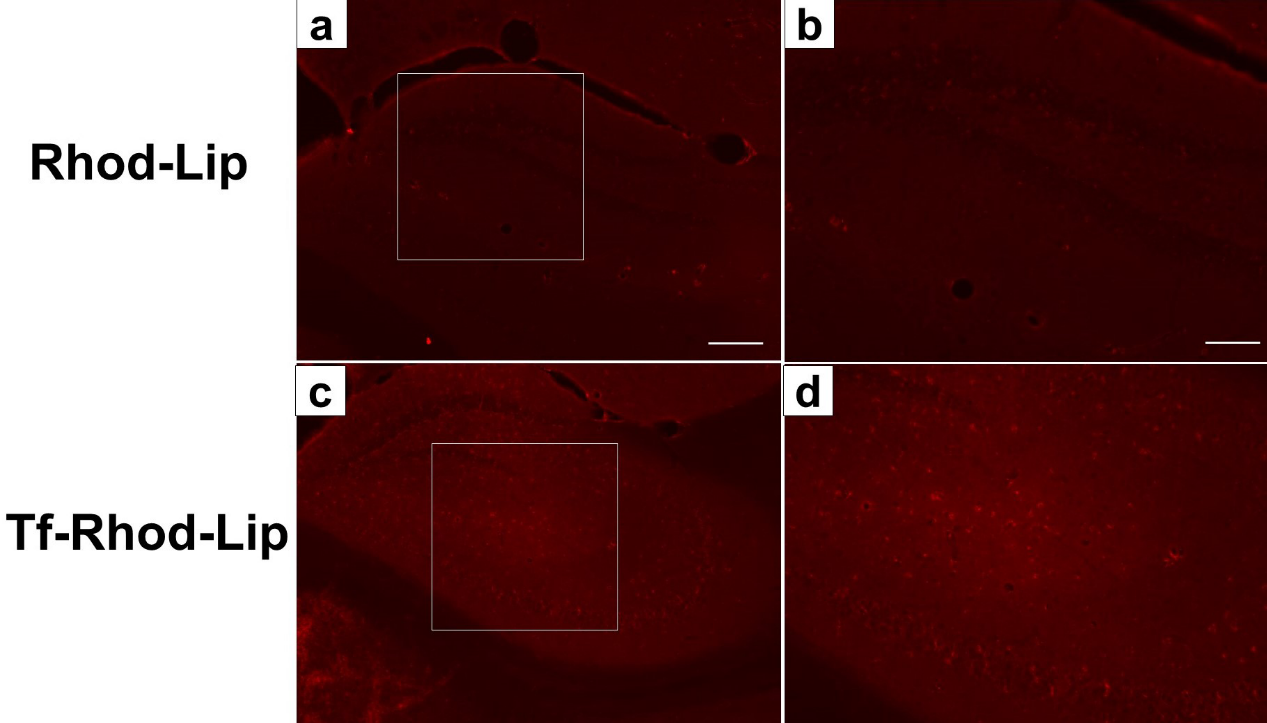


**Figure S2 Biodistribution of rhodamine-labeled and transferrin-modified liposomes (Tf-Rhod-Lip) in the hippocampus of mice.** Rhodamine signals, followed for up to 3 days’ administration with 5 mg lipids/kg Rhod-Lip (a-b) and Tf-Rhod-Lip (c-d), are detected in the dorsal hippocampal regions. b and d are magnifications of the framed areas of a and c. Scale bar, 200 μm (to the left panels) and 100 μm (to the right panels).


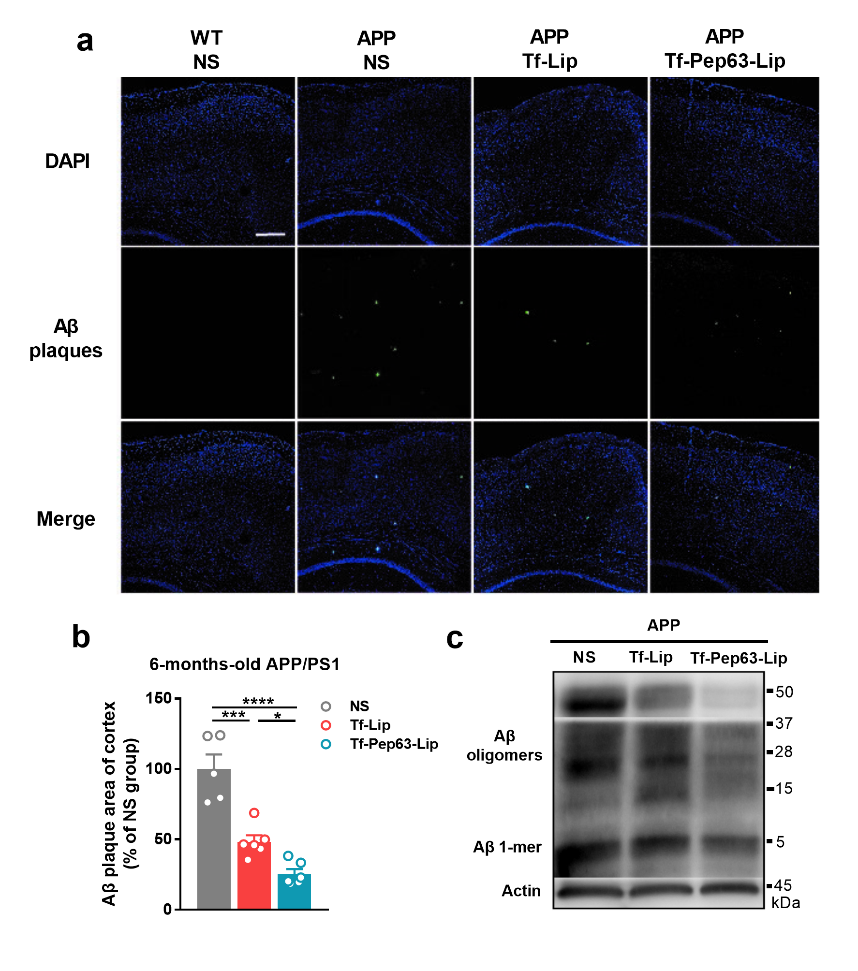


**Figure S3 Tf-Pep63-Lip and Tf-Lip reduce Aβ1-42 levels in APP/PS1 mice.** **a** Representative immunofluorescence images of Aβ1-42 plaques (green) in the cortex of APP/PS1 mice treated with NS, Tf-Lip, or Tf-Pep63-Lip at the lipids dose of 5 mg/kg intravenously. **b** The area of Aβ1-42 plaques is quantified and normalized to NS-treated APP/PS1 mice. Tf-Lip and Tf-Pep63-Lip significantly decrease the area of the Aβ1-42 plaque in the cortex of APP/PS1 mice (*n* = 6). Scale bar: 200 µm. **P* < 0.05, ****P* < 0.001, *****P* < 0.0001, significantly different. Data are presented as means ± SEM. **c** Representative Western blot of hippocampal soluble Aβ probed with 6E10 after treatment of NS, Tf-Lip, or Tf-Pep63-Lip.


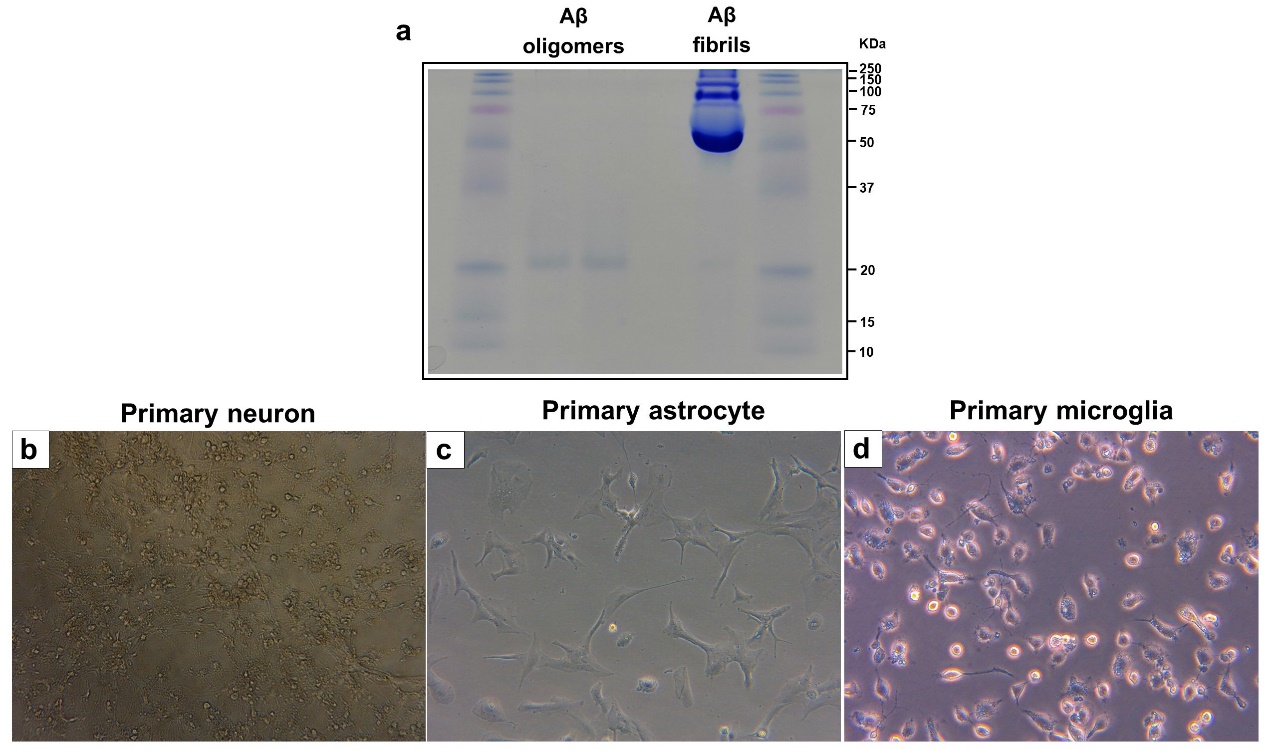


**Figure S4 Identification of prepared Aβ aggregations and primary cells.** **a** The bands of synthetic Aβ oligomers and fibrils in SDS-PAGE gels by Coomassie blue staining. **b-d** Morphology of cultured primary neuron, astrocyte and microglia by the phase contrast microscope.
